# Draft Genome Sequence of an Obligate and Moderately Halophilic Bacterium, *Thalassobacillus devorans* Strain MSP14, the First Draft Genome of the Genus *Thalassobacillus*

**DOI:** 10.1128/genomeA.01103-13

**Published:** 2013-12-26

**Authors:** Kamal Krishna Pal, Rinku Dey, Dharmesh Sherathia, Bhoomika Sukhadiya, Trupti Dalsania, Ilaxi Patel, Kinjal Savsani, Manesh Thomas, Sejal Vanpariya, Mona Mandaliya, Rupal Rupapara, Priya Rawal, Sucheta Ghorai, Sharmila Bhayani, Abhi Shah, Anil Kumar Saxena

**Affiliations:** Microbiology Section, Directorate of Groundnut Research, Junagadh, Gujarat, Indiaa; Division of Microbiology, Indian Agricultural Research Institute, New Delhi, Indiab

## Abstract

We report the 3.93-Mbp first draft genome sequence of a species of the genus *Thalassobacillus*, *Thalassobacillus devorans* strain MSP14, a moderate but obligate halophile, isolated from a salt crystallizer of the Little Rann of Kutch, India. Exploring the genome of this organism will facilitate understanding the mechanism(s) of its obligate halophilism.

## GENOME ANNOUNCEMENT

The genomes of a number of halophilic bacilli, isolated from the salt crystallizers of the Rann of Kutch, Gujarat, India, have been sequenced with a view to understanding the mechanism(s) of osmotolerance (1–4). *Thalassobacillus devorans* strain MSP14 (16S rRNA, GenBank accession no. JX518269), an obligate but moderately halophilic bacterium, was isolated from a salt crystallizer of the Little Rann of Kutch, India. It grows optimally at a concentration of 7.5% NaCl (range, 5 to 15%) in medium at 37°C and pH 7.5. The present genome of MSP14 was sequenced to understand the mechanism(s) of obligate, but moderate, halophilism.

By use of the Roche 454 genome sequencer (GS FLX), the genome of *Thalassobacillus devorans* strain MSP14 (G+C content of 42.97%) was sequenced at Macrogen, Inc., South Korea, through Sequencher Tech Pvt., Ltd., Ahmedabad, India. Both shotgun and 3-kb mate-paired library sequencing were performed. Whereas sequencing of shotgun libraries generated 787,155 reads of 439,717,712 bases (average read length of 558 bp), mate-paired libraries generated 140,683 and 131,751 reads of 62,893,040 and 57,724,648 bases, respectively, with average read lengths of 447 and 438 bp, respectively.

*De novo* assembly of the reads using GS De Novo Assembler v 2.6 (5) gave approximately 138-fold coverage with 9 scaffolds of 3,935,133 bp and 32 contigs of 3,923,021 bp with average lengths of 437,237 bp and 122,594 bp, respectively. An *N*_50_ scaffold length of 1,214,989 bp (smallest, 2,007 bp and largest, 1,848,831 bp) and an *N*_50_ contig length of 260,182 bp (smallest, 1,596 bp and largest 467,343 bp) were obtained. All assembly data were deposited in the DDBJ/EMBL/GenBank nucleotide sequence database.

The draft genome was annotated by use of the RAST server (6), Glimmer 3 (7, 8), GeneMark (9, 10), the KEGG database (11), tRNAScan-SE (12), RNAmmer (13), and Signal P4.1 (14).

Using the different software tools, we predicted 4,011 coding sequences (CDS), 76 RNA-encoding genes (72 tRNA and 4 rRNA), 464 subsystems, and 31 signal peptides. Among the CDS, 2,097 are not in a subsystem (955 nonhypothetical and 1,142 hypothetical), whereas 1,914 CDS (1,819 nonhypothetical and 995 hypothetical) are in a subsystem. RAST annotation also revealed the association of 123 genes in stress responses: 25 genes in osmotic stress (1 in osmoregulation, 4 in ectoine biosynthesis and regulation, and 20 in choline and betaine uptake and betaine biosynthesis), 44 in oxidative stress (8 in protection from reactive oxygen species [ROS], 21 in oxidative stress, 1 in the glutathione: redox cycle, 7 in redox-dependent regulation of nuclear processes, 2 in glutathione: nonredox reactions, 2 in glutaredoxins, 1 in the CoA disulfide thiol-disulfide redox system, and 2 in cluster-containing glutathione synthetase), 4 in cold shock, 17 in heat shock, 9 in detoxification, and 24 in no subcategory. Similarly, 1,840 genes were mapped to different biochemical pathways of KEGG (map00010 to map05340), including genes in ABC transporters (map02010) and two-component systems (map02020) like transporters for glycine betaine/proline (ProX, ProW, and ProV), osmoprotectants (OpuBC and OpuBA), phosphates (PstA, PstB, PstC, and PstS), salt stress-degradative enzymes (DegS and DegU), etc.

We are exploring the genome of *Thalassobacillus devorans* MSP14 further to unravel the mechanism(s) of its obligate halophilism.

### Nucleotide sequence accession numbers.

This whole genome shotgun project has been deposited at DDBJ/EMBL/GenBank under the accession number AWXW00000000. The version described in this paper is version AWXW01000000.
